# Anthelmintic activity of *Indigofera tinctoria* against gastrointestinal nematodes of sheep

**DOI:** 10.14202/vetworld.2016.101-106

**Published:** 2016-01-31

**Authors:** Ambalathaduvar Meenakshisundaram, Tirunelveli Jayagopal Harikrishnan, Thavasi Anna

**Affiliations:** 1 Department of Veterinary Parasitology, Veterinary College and Research Institute, Tirunelveli - 627 358, Tamil Nadu, India; 2 Registrar, Tamil Nadu Veterinary and Animal Sciences University, Chennai - 600051, Tamil Nadu, India

**Keywords:** anthelmintic, evaluation, gastrointestinalnematodes, *Indigofera tinctoria*, sheep

## Abstract

**Aim:**

Gastrointestinal (GI) nematodes are considered as a major constraint for successful sheep production. Control of these parasites heavily relies on the use of chemical anthelmintics. Over the past decades, the development of anthelmintic resistance to various groups of anthelmintics and problem of drug residues in animal products has awakened interest in medicinal plants as an alternative source of anthelmintics. Hence, this study was undertaken to evaluate the anthelmintic efficacy of *Indigofera tinctoria* by scientifically validated *in vitro* and *in vivo* tests approved by the World Association for the Advancement of Veterinary Parasitology.

**Materials and Methods:**

*In vitro* assays such as egg hatch assay for ovicidal and larval migration inhibition and larval development assay for larvicidal properties were used to investigate *in vitro* effect of extracts on strongyle egg and larvae, respectively. Fecal egg count reduction test was conducted *in vivo* to evaluate the therapeutic efficacy of the extracts administered orally at dose rates of 125, 250, 500 mg/kg to sheep naturally infected with mixed GI nematodes.

**Results:**

Ethanolic extract of *I. tinctoria* demonstrated significant (p<0.01) inhibition on egg hatching at concentrations of 40 mg/ml and 80 mg/ml. In *in vivo* assay, the ethanolic extract of *I. tinctoria* reduced the fecal egg count ranging between 30.82% and 47.78% at various doses (125, 250 and 500 mg/kg). Although there was a slight variation, all the hematological parameters were within the normal range reported for sheep. Except for alanine transaminase, the overall mean of all the serum biochemical profile was within the normal range for sheep.

**Conclusion:**

Based on the results obtained by *in vitro* and *in vivo* assay, the ethanolic extract of *I. tinctoria* possesses anthelmintic activity and could replace the chemical anthelmintics used presently.

## Introduction

Sheep production plays a vital role in augmenting socio-economic status of the rural masses, particularly the small landholders and landless farmers, who rely on these animals for their animal protein source and income for their livelihood [1]. However, mismanagement, poor hygiene, and precarious housing conditions all contributed to the incidence of disease and high mortality. Parasitic diseases, especially gastrointestinal (GI) nematodes, are among the factors that limit sheep production worldwide, accounting for thelargest economic losses due to retarded growth, weight loss, reduced food consumption, lower milk production, impaired fertility, and in cases of massive infections, highmortality rates [2].

Currently, nematode control programs in sheep depend mainly through the use of anthelmintics. Development of anthelmintic resistance [3], increased public awareness over the drug residues in animal products and toxicity problems [4] has necessitated an intensified effort to find alternative endoparasite control measures that are both feasible and economical for the farmers [5]. Among the alternative strategies, there has been considerable and expanding interest in traditional herbal dewormers in both developed and developing countries. Several studies have showed that plant species can effectively reduce the degree of parasite infestation in livestock and are promising alternatives to conventional anthelmintics [6,7] that are both sustainable and environmentally acceptable.

Hence, the present study was envisaged to evaluate the anthelmintic properties of *Indigofera tinctoria* plant extracts by scientifically validated *in vitro* and *in vivo* techniques.

## Materials and Methods

### Ethical approval

The study was conducted after the approval of the Institutional Animal Ethics Committee.

### Collection of plant materials and extraction

*I. tinctoria* is a medicinal plant belonging to the family Papilionaceae and extensively used for its blue dye indigo. It is a small erect shrub cultivated in all parts of India, especially in the Southern India, Tamil Nadu. The plant is a purgative, antiseptic and astringent. In Ayurveda and Siddha systems, the entire plantsareused for the treatment of helminthic infections. The leaves of *I. tinctoria* were collected from the local field and certified by a botanist. The collected plant materials were shade dried, powdered and stored in airtight container for further extraction. The aqueous extract was prepared as prescribed by Onyeyili *et al*. [8]. The dried extract wascollected in stoppered vials and stored at 4°C until use. The ethanolic extract was prepared as described by Wang and Waller [9].

### Gas chromatography–mass spectrometry (GC-MS)analysis

GC-MS analysis was carried out on a GC clarus 500 Perkin Elmer system comprising an AOC-20i autosampler and GC-MS instrument employing the following conditions: Column Elite-1 fused silica capillarycolumn (30 mm ID × 0.25 mm ID × 1 µM df), composed of 100% dimethyl poly diloxide, operating in electron impact mode at 70eV; helium (99.99%) was used as carrier gas at a constant flow of 1 ml/min and an injection volume of 0.5 µl was employed (split ratio of 10:1) injector temperature 250°C; ionsource temperature 280°C. The oven temperature was programed from 110°C (isothermal for 2 min), with an increase of 10°C/min, to 200°C, then 5°C/min to 280°C, ending with a 9 min isothermal at 280°C. Mass spectra were taken at 70 eV; a scan interval of 0.5 s and fragments from 40 to 450 Da. Total GC running time is 36 mininterpretation on mass spectrum GC-MS was conducted using the database of the National Institute Standard and Technology (NIST) having more than 62,000 patterns. The spectrum of the unknown component was compared with the spectrum of the known components stored in the NIST library. The name, molecular weight and structure of the components of the test materials were ascertained.

### Preparation plant/drug stock solution

Pure thiabendazole and levamisole (0.1 g, Sigma, USA) were transferred into a 100 ml volumetric flask through a small glass funnel and rinsed twice, each with 10 ml of dimethyl sulfoxide (DMSO). A further 20 ml of DMSO was added, and the total volume was made up to 100 ml with distilled water. Using the stock solution, a suitable working solution of thiabendazole with a final concentration of 200 µg/ml was prepared and used as positive control. Stock solutions of crudeaqueousandethanolicextracts of *I. tinctoria* initially were prepared by dissolving the crude extracts in DMSO so as to improve their solubility in water. Aliquots of stock solution (100 mg/ml) were further diluted to obtain final concentrations of 10 (1%), 20 (2%), 40 (4%), and 80.0 (8%) mg/ml for each extract.

### In vitro tests

Plant extracts at concentrations of 10, 20, 40 and 80 mg/ml were used for all *in vitro* assays.

Egg hatch assay - Pooled fecal samples were obtained by mixing several samples collected per rectum from a number of sheep naturally infected with mixed GI nematodes. About 40 ml of water was added to the fecal sample and kneaded thoroughly. The macerated fecal material was then suspended in 1 L of tap water and the fecal suspension was washed over a series of sieves of decreasing sizes (500, 75 and 35 µm). The retentate in the 35 µm sieve, which contained the nematode eggs was washed and collected in a polyallomer tube and centrifuged at 1000 rpm for 1-2 min. After removing the supernatant, the sediment was resuspended in 10-12 ml of saturated sodium chloride solution. After thorough and gentle mixing, the suspension was centrifuged again at 1000 rpm for 1-2 min. Using artery forceps, the polyallomer tube was clamped just below the meniscus and the contents above the clamp were transferred into a 15 ml polystyrene tube and washed twice with distilled water. Isolated eggs were pooled and made up to a volume of 10 ml. From this suspension, 100 µl was pipetted eggs counted and resuspended in such a manner that 100 µl of the suspension contained approximately 100 eggs. The assay was performed in 24 well plates as per the method described by Jackson *et al*. [10]. The percentage of hatch for each concentration was calculated, and the results were subjected to probit analysis to obtain effective dose 50% values.

In larval development assay, eggs were harvested from the pooled fecal samples and the concentration of eggs was estimated in 100 µl samples and adjusted to 100 eggs per 100 µl. The assay was conducted as per the methods designed by Hubert and Kerboeuf [11]. The mean larval development for each drug concentration and the lethal dose 50% value was determined by plotting the percentage larval development and drug concentration.

Larval migration assay was conducted as per the method described by Jackson *et al*. [10]. The number of larvae retained by the mesh (Nr) and those that had migrated (Nm) through the mesh was counted. The drug concentration against percentage migration was plotted over a graph and the lethal mutation 50% values were derived.

### In vivo tests

Vembur sheep maintained under semi-intensive system of management at Instructional Livestock Farm Complex, Veterinary College and Research Institute, Ramayanpatti, Tirunelveli were utilized for this study. 30 vembur lambs of 6-12 months age which showed eggs per gram of feces (EPG) from 1000 to 2700 before treatment were selected and randomly distributed into five treatment groups each comprising of six animals. Three groups were treated with doses of plant extracts at 125, 250 and 500 mg/kg, respectively, while the fourth and the fifth group served as positive and negative controls. Fecal samples were collected from each animal on day 0 and at day 12 post-treatment and fecal egg count reduction (FECR) was assessed as recommended by the World Association for the Advancement of Veterinary Parasitology [12].

### Estimation of hematological and serum parameters

Blood samples were collected on day 0, 3, and 12 post-treatment from each animal and hematological parameters were determined as described by Jain [13]. Serum biochemical profiles were determined using standard diagnostic kits obtained from Span Diagnostics Ltd. Pooled fecal samples were cultured and identified as described by MAFF [14].

### Statistical analysis

For *in vitro assays*, probit transformation was performed to transform a typical sigmoid dose-response curve to linear function [11]. Fecal egg count, hematological and serum biochemical parameters were analyzed by the statistical methods as described by Snedecor and Cochran [15].

## Results

The results of *in vitro* assays were shown in Table-1. Of the four doses (10, 20, 40 and 80 mg/ml) tested, ethanolic extract of *I. tinctoria* induced a significant egg hatch inhibition at 40 and 80 mg/ml and aqueous extract induced only marginal inhibition at all the concentrations tested. On the other hand, both aqueous and ethanolic extracts of *I. tinctoria* did not induce significant inhibition of larval development andmigration. Only, ethanolic extract of *I. tinctoria* which was proved to be effective *in vitro* egg hatch assay was further evaluated by *in vivo* assay (FECR test).

**Table-1:** Mean percent efficacy of aqueous and ethanolic extracts of *I. tinctoriaon* nematode egg hatch, larval migration and larval development inhibition.

Name of the plant extracts	Concentration of the plant extracts				Positive control	Negative control

	1%	2%	4%	8%		
Egg hatch assay						
Aqueous extract	7.50^c^±0.35	12.50^bc^±0.35	18.50^ab^±0.35	24.50^a^±4.50	97.03^a^±0.82	8.79^d^±1.05
Ethanolic extract	50.00^c^±8.00	61.00^b^±1.00	68.50^ab^±0.50	73.50^a^±0.50	97.03^a^±0.82	8.79^b^±1.05
Larval migration inhibtion assay						
Aqueous extract	25.60^b^±4.17	34.09^b^±3.16	30.20±2.20	34.52±5.11	96.22^a^±0.35	8.27^c^±0.98
Ethanolic extract	5.78^c^±1.19	8.15^bc^±0.65	15.10^a^±1.78	14.40^a^±1.47	96.22^a^±0.35	8.27^c^±0.98
Larval development assay						
Aqueous extract	12.50±0.90	13.50±3.10	19.80±2.00	17.70±1.10	93.46^a^±1.82	5.30^b^±1.12
Ethanolic extract	20.20±3.40	18.90±1.70	21.60±3.60	22.30±2.10	93.46^a^±1.82	5.30^d^±1.12

Values sharing any one common superscript in a row (overall) do not differ significantly (p<0.01). NS=Not significant, *I. tinctoria=Indigofera tinctoria*

The percentage reduction in fecal egg counts of sheep treated with different doses of ethanolic extract of *I. tinctoria* and albendazole are presented (Table-2). The ethanolic extract of *I.tinctoria* produced a dose-dependent reduction in EPG on 12 days post-treatment with higher reduction of 47.78% at 500 mg/kg. Sheep drenched with albendazole (albomar - positive control) at 7.0 mg/kg showed 93.25% reduction in EPG.

**Table-2:** Effect of *I. tinctoria* ethanolic extracton percent FECR (mean±SE) in sheep naturally infected with GI nematodes.

Dose (mg/kg)	Ethanolic extract of *I. tinctoria*	Albendazole (7 mg/kg)
125	30.82^cB^±1.90	93.25^a^±1.15
250	41.41^bA^±2.06	93.25^a^±1.15
500	47.78^bA^±2.04	93.25^a^±1.15

(*P*<0.01) Values sharing any one common superscript in a row (small letters) and column (capital letters) do not differ significantly. *I. tinctoria=Indigofera tinctoria*, FECR=Fecal egg count reduction, GI=Gastrointestinal, SE=Standard error

### Coproculture

*Oesophagostomum columbianum* was the primary GI nematode infecting animals with 71%. *Haemonchus contortus* was the second averaging 27% followed by *Bunostomum* spp. (1%).

### Effect of plant extracts on hematological values of sheep

The mean values of hemogram and serum profilein sheep treated with ethanolic extract of *I. tinctoria* and albendazole (albomar) (positive control) are presented (Tables-3 and 4). There was not much variation in hemogram and serum biochemical levels before and after treatment at all the doses tested.

**Table-3:** Effect of *I. tinctoria* ethanolic extracton blood parameters (mean±SE) in sheep naturally infected with GI nematodes.

Serum parameters	Period	Dose (mg/kg)			Positive control	Negative control

		125	250	500		
PCV (%)	0-day	29.33±0.71	28.90±0.25	29.47±0.58	26.73±2.01	27.47±2.11
	3^rd^-day	25.00±4.36	30.10±4.97	36.00±4.51	27.77±2.10	28.07±1.65
	12^th^-day	22.00±10.54	26.67±1.76	27.67±1.67	29.00±2.45	29.07±1.77
	Overall	25.44^bcd^±3.47	28.56^ab^±1.61	31.04^a^±1.89	27.83^abc^±1.14	28.20^ab^±0.95
Hb concentration (g/dL)	0-day	9.70±0.29	9.87±0.12	9.97±0.17	9.33±0.73	9.57±0.88
	3^rd^-day	9.50±0.75	10.73±1.45	10.37±0.24	9.77±0.74	9.97±0.86
	12^th^-day	11.17±1.57	10.57±1.19	10.30±0.29	10.13±0.78	10.23±0.88
	Overall	10.12^b^±0.57	10.39^a^±0.56	10.21^ab^±0.13	9.74^abcd^±0.39	9.92^abc^±0.45
TEC (10^6^/μl)	0-day	8.88±0.34	8.98±0.13	9.02±0.20	8.63±0.76	8.47±0.75
	3^rd^-day	9.10±0.35	9.08±0.16	9.17±0.33	8.76±0.76	8.69±0.80
	12^th^-day	9.20±0.25	8.86±0.13	9.20±0.21	8.96±0.76	9.07±0.72
	Overall	9.06^ab^±0.17	8.97^abc^±0.08	9.13^a^±0.13	8.78^abcde^±0.38	8.74^abcde^±0.39
TLC (10^3^/μl)	0-day	8.80±0.17	8.77±0.24	8.17±0.81	6.77±0.52	8.67±0.79
	3^rd^-day	9.10±0.21	9.10±0.21	8.43±0.84	7.60±0.25	9.33±0.66
	12^th^-day	9.00±0.12	8.97±0.19	8.40±0.64	8.00±0.21	9.23±0.33
	Overall	8.97^ab^±0.10	8.94^bc^±0.12	8.33^d^±0.39	7.46^e^±0.25	9.08^a^±0.33

Values sharing any one common superscript in a row (overall) do not differ significantly (p<0.01). NS=Not significant (p<0.05), PCV=Packed cell volume, TEC=Total erythrocyte count, TLC=Total leukocyte count, *I. tinctoria=Indigofera tinctoria,* GI=Gastrointestinal, SE=Standard error

**Table-4:** Effect of *I. tinctoira* ethanolic extract on serum parameters (mean±SE) in sheep naturally infected with GI nematodes.

Serum parameters	Period	Dose (mg/kg)			Positive control	Negative control

		125	250	500		
BUN	0-day	49.07±3.50	48.77±0.26	50.77±1.82	43.37±1.78	47.23±6.89
	3^rd^-day	38.90±1.85	38.93±1.10	50.70±3.00	52.90±2.25	42.47±11.11
	12^th^-day	48.17±5.74	48.70±1.46	49.33±2.03	47.10±1.46	49.43±1.15
	Overall	45.38^abcd^±2.59	45.47^abcd^±1.72	50.27^ab^±1.19	47.79^abcd^±1.67	46.38^abcd^±3.93
Serum creatinine	0-day	1.47±0.12	1.37±0.03	1.53±0.03	1.40±0.06	1.40±0.12
	3^rd^-day	1.30±0.00	1.33±0.03	1.47±0.07	1.53±0.09	1.57±0.09
	12^th^-day	1.50±0.12	1.47±0.03	1.53±0.03	1.43±0.03	1.47±0.03
	Overall	1.42^bc^±0.06	1.39^bc^±0.03	1.51^abc^±0.03	1.46^bc^±0.04	1.48^bc^±0.05
SGOT	0-day	115.83±2.89	103.23±2.57	135.13±10.87	124.67±2.11	109.20±12.01
	3^rd^-day	106.67±8.70	115.67±14.28	125.43±7.97	103.97±3.98	97.53±12.16
	12^th^-day	121.63±7.86	96.77±5.62	116.70±9.50	113.47±17.02	90.27±5.49
	Overall (NS)	114.71±4.11	105.22±5.28	125.76±5.45	114.03±5.90	99.00±5.87
SGPT	0-day	29.00±2.30	20.90±4.08	13.40±3.38	27.83±5.98	20.70±1.88
	3^rd^-day	24.40±3.92	30.00±3.25	23.03±6.93	23.40±3.80	18.90±3.09
	12^th^-day	18.13±2.10	21.70±2.15	20.97±0.45	21.10±1.00	17.00±0.61
	Overall (NS)	23.84±2.14	24.20±2.18	19.13±2.67	24.11±2.29	18.87±1.19

Values sharing any one common superscript in a row (overall) do not differ significantly (p<0.01). NS=Not significant (p<0.05), SGPT=Serum glutamic pyruvic transaminase, SGOT=Serum glutamicoxaloacetic transaminase, BUN=Blood urea nitrogen, *I. tinctoria=Indigofera tinctoria*, GI=Gastrointestinal, SE=Standard error

### Phytocomponents

**Figure-1:**
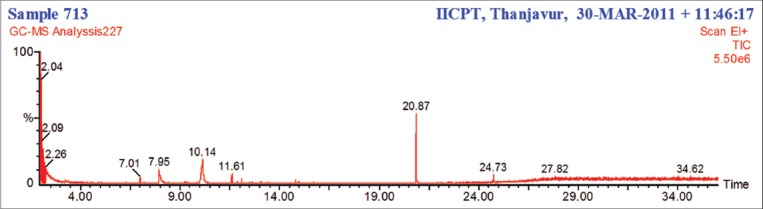
Gas chromatography-mass spectrometry chromatogram of the ethanolic extract of Indigofera tinctoria.

About 10 phytocomponents were identified in the phytochemical screening of the ethanolic extract of *I. tinctoira* (Table-5 and Figure-1).

**Table-5:** Phytocomponents identified in the ethanolic extract of *I. tinctoira*.

RT	Name of the compound	Molecular formula	MW	Peak area %
7.01	1,3,5-Triazine-2,4,6 (1H,3H,5H)trione	C_3_H_3_N_3_O_3_	129	2.31
7.95	Hydrazine, 1,2-dimethyl-	C_2_H_8_N_2_	60	13.33
10.14	Myo-Inositol, 2-Cmethyl-	C_7_H_14_O_6_	194	30.00
11.61	2-Tetradecyne	C_14_H_26_	194	1.54
12.10	Z-2-Dodecenol	C_12_H_24_O	184	0.51
13.07	Ethaneperoxoic acid, 1-cyano-1-[2-(2-phenyl-1,3-dioxolan-2-yl) ethyl] pentyl ester	C_19_H_25_NO_5_	347	0.26
14.83	3-Butyn-2-ol	C_4_H_6_O	70	0.51
14.96	Orthoformic acid, tri-2-butenyl ester	C_13_H_22_O_3_	226	0.26
20.87	1,2-Benzenedicarboxylic acid, diisooctyl ester	C_24_H_38_O_4_	390	25.64
24.73	1,5-Heptadiene, 2,6-dimethyl-	C_9_H_16_	124	25.64

*I. tinctoria=Indigofera tinctoria*, MW=Molecular weight

## Discussion

Ethanolic extract of *I. tinctoria* demonstrated significant (p<0.05) inhibition of egg hatching at 40 and 80 mg/ml. Increasing concentration of the plant extracts resulted in increased inhibition of egg hatching indicating dose-dependent activity. Similar dose-dependent *in vitro* egg hatching inhibition was evaluated using aqueous extract of *Caryocar brasiliense* camb [16] and aqueous and methanolic extract of *Ocimum sanctum* [17] against *H. contortus*. The findings are in agreement with Balamurugan and Selvarajan [18] who reported that the methanolic extract of *I. tinctoria* revealed maximum activity against the Indian earthworm *Pheretima posthuma* owing to its anatomical and physiological ­resemblance with the intestinal roundworm of humans.

Both the extracts of *I*. *tinctoria* inhibited migration of *H. contortus* larvae only to the level of 5.78-34.52%. In contrast, Bendixsen *et al*. [19] reported larvicidal activity by LMIA with aqueous extracts obtained from *Caliandra* spp., *Leucaena glauca* and *Acacia farnesiana* at 0.8 mg/ml.

Larval development was not inhibited by both aqueous and ethanolic extracts of *I. tinctoira* which was comparable with the results recorded by Ademola *et al*. [20] when aqueous and ethanolic extracts of *Nauclea latifolia* were used.

The highest percent FECR (47.78%) recorded with ethanolic extract of *I. tinctoria* was in agreement with Soro *et al*. [21] who recorded a 81% FECR at a single oral dose of 80 mg/kg 3 weeks post-treatment using ethanolic extract of *Anogeissus leiocarpus* in sheep naturally infected with GI nematodes. The results were in par with the findings recorded by Mesquita Mde and Batista [22] with *Eucalyptus staigeriana* essential oil, banana crop residues [23] and *Lespedeza cuneata* [24].

The observed increase in the hemoglobin (Hb) levels in animals was in accordance with the findings of Hossain *et al*. [25] who reported increased Hb content in sheep when treated with neem leaves and which might be due to increased absorption of iron. Similar increase in Hb and total erythrocyte count were also recorded by Rahman [26] with neem, betel leaf and jute leaves in goats and Rob *et al*. [27] with aqueous extract of neem leaves in sheep. The increase in total leukocyte count in the present study was in accordance with the result obtained by Agaie and Onyeyili [28] with *A. leiocarpus* which might probably be due to the immune response to infection or sensitization.

Blood urea nitrogen and serum creatinine values were within normal in treated animals which implies that plant extracts used in the current study have no adverse effects on the kidney. Higher serum alanine transaminase values above normal in this study as recorded by Ajala *et al*. [29] with *Millettia thonningii* leaves in bucks, gradually decreased during the observation period which is comparable to values recorded in the control animal group. This implies that these extracts have no toxic effects on liver and heart.

Phytochemical analysis of the ethanolic extract of *I. tinctoira* revealed that the mechanism of action is not fully understood. However, the collective or individual presence of bioactive compounds in the extract may possibly constitute the basis for the profound anthelmintic activity exhibited by the plant extract as opined by Ruben *et al*. [30].

## Conclusion

Ethanolic extract of *I. tinctoira* possess potential anthelmintic activity and offer an alternative source for the control of GI nematodes of sheep.

## Authors’ Contributions

TJH designed the experiment, sample collection and experiment was performed by AM under the supervision of TJH. Manuscript preparation was supervised, reviewed and edited by TJH and TA. All authors read and approved the final manuscript.
